# Species composition of sand flies and bionomics of *Phlebotomus papatasi* and *P. sergenti* (Diptera: Psychodidae) in cutaneous leishmaniasis endemic foci, Morocco

**DOI:** 10.1186/s13071-016-1343-6

**Published:** 2016-02-02

**Authors:** Samia Boussaa, Kholoud Kahime, Abdallah M. Samy, Abdelkrim Ben Salem, Ali Boumezzough

**Affiliations:** Laboratory of Ecology and Environment (URAC 32, CNRST; ERACNERS 06), Faculty of Sciences Semlalia, Cadi Ayyad University, Marrakesh, Morocco; Institut Supérieur des Professions Infirmières et des Techniques de Santé (ISPITS), Ministry of Health, Marrakesh, Morocco; Entomology Department, Faculty of Science, Ain Shams University, Cairo, 11566 Egypt; Laboratory of Hydrobiology, Ecotoxicology and Sanitation (LHEA), Faculty of Sciences Semlalia, Cadi Ayyad University, Marrakesh, Morocco

**Keywords:** *Phlebotomus papatasi*, *P. sergenti*, Temperature, Soil, NDVI, Environmental factors, Cutaneous leishmaniasis, Morocco

## Abstract

**Background:**

Cutaneous Leishmaniasis (CL) is one of the most neglected tropical diseases in Morocco. *Leishmania major* and *L. tropica* are the main culprits identified in all endemic foci across the country. These two etiological agents are transmitted by *Phlebotomus papatasi* and *P. sergenti*, the two most prevalent sand fly species in Morocco. Previous studies reflected gaps of knowledge regarding the environmental fingerprints that affect the distribution of these two potential vectors across Morocco.

**Methods:**

The sand flies were collected from 48 districts across Morocco using sticky paper traps. Collected specimens were preserved in 70 % ethanol for further processing and identification. Male and female densities were calculated in each site to examine their relations to the environmental conditions across these sites. The study used 19 environmental variables including precipitation, aridity, elevation, soil variables and a composite representing maximum, minimum and mean of day- and night-time Land Surface Temperature (LST), and Normalized Difference Vegetation Index (NDVI).

**Results:**

A total of 11,717 specimens were collected during this entomological survey. These specimens represented 11 species of two genera; *Phlebotomus* and *Sergentomyia*. Correlations of the sand fly densities with the environmental variables were estimated to identify the variables which influence the distribution of the two potential vectors, *Phlebotomus papatasi* and *P. sergenti*, associated with all CL endemic foci across the country. The density of *P. papatasi* was most affected by temperature changes. The study showed a significant positive correlation between the densities of both sexes of *P. papatasi* and night-time temperatures. Both *P. papatasi* and *P. sergenti* showed a negative correlation with aridity, but, such correlation was only significant in case of *P. papatasi*.

NDVI showed a positive correlation only with densities of *P. sergenti*, while, soil PH and soil water stress were negatively correlated with the densities of both males and females of only *P. papatasi*.

**Conclusions:**

Our results identified the sand fly species across all CL endemic sites and underlined the influences of night-time temperature, soil water stress and NDVI as the most important variables affecting the sand fly distribution in all sampled sites. This preliminary study considered the importance of these covariates to anticipate the potential distribution of *P. papatasi* and *P. sergenti* in Morocco.

**Electronic supplementary material:**

The online version of this article (doi:10.1186/s13071-016-1343-6) contains supplementary material, which is available to authorized users.

## Background

Cutaneous and visceral leishmaniasis (kala-azar) represent the most neglected tropical disease across the world [1]. In Morocco, cutaneous leishmaniasis (CL) is endemic and constitutes a major public health threat [2]. CL is widely distributed in three nosogeographic entities across Morocco; zoonotic cutaneous leishmaniasis (ZCL; caused by *L. major*) located in the arid regions along the northern edge of the Sahara desert, anthroponotic cutaneous leishmaniasis (ACL; caused by *L. tropica*) in the semi-arid regions of central and south-western Morocco, and CL caused by *L. infantum* in the northern regions of the country [3, 4].

Moroccan Ministry of Health has reported 24,804 cases of ZCL and 16,852 cases of ACL in 2004–2013 alone [5]. CL caused by *L. infantum* remains a very rare condition with few sporadic cases occurring, especially in northern Morocco [4]. Even with the high number of cases reported during recent years, leishmaniasis remains underestimated, especially in remote and rural communities where there is no access to health facilities. CL is the least known and most neglected disease, especially among males in all endemic areas in Morocco: males mostly depend on traditional medicine which becomes overpowered in the endemic areas. Lack of awareness among males reflected the higher incidence of leishmaniasis occurred among females and children [6].

ZCL transmission was maintained for a long term in rodent reservoirs, for example, *Meriones shawi grandis* is recognized as the main mammalian reservoir host to maintain local circulation of *L. major* [7] . On the other hand, human was identified as the only reservoir host for ACL caused by *L. tropica* [4]; however, zoonotic transmission for this species was also reported from other countries where the parasite was isolated from rock hyrax, and gerbil in Israel and Egypt, respectively [8, 9].

*Leishmania* infection is transmitted locally to a human host by the bite of an infected female sand fly (Diptera: Psychodidae) of the genus *Phlebotomus. Phlebotomus* (*Phlebotomus*) *papatasi* Scopoli, 1786 and *P*. (*Paraphlebotomus*) *sergenti* Parrot, 1917 were identified as the only proven vectors for ZCL and ACL, respectively [10, 11].

Over the past decade, the epidemiological situation of CL has changed significantly in Morocco; the disease was reported and geographically expanded to new habitats which were never reported as leishmaniasis endemic foci. This rapid expansion of CL in the country increased the public health problems associated with the disease epidemics. Previous studies revealed that this expansion in the disease dynamics may be associated with range expansions of the vector populations in response to climate change [12, 13].

*P. papatasi* and *P. sergenti* were found highly dependent on environmental conditions [14–16]. Environmental factors may affect several ecological and biological processes that act directly on controlling the vector’s geographical distribution, abundance and reproductive rates. All of these associations between environmental conditions and the distribution of sand fly vectors reveal a gap of knowledge to the current situation in Morocco; however, evidences are available from other countries in the region [17, 18].

This current contribution is a preliminary analysis to update the sand fly species composition across Morocco and examine the relationship between the potential distribution of *P. papatasi* and *P. sergenti* in the region in response to climatic (i.e. temperature, aridity and precipitation), topographic, soil and the greenness variables; taking advantage of the recent high resolution satellite data available across the study area. The preliminary results presented here are the first set of analysis to evaluate the importance of multidimensional factors in limiting the distribution of two potential CL vectors. They represent a baseline for more detailed studies across the country to better understand the ecology of sand fly vectors and guiding the national control program.

## Methods

### Study area

**Fig. 1:**
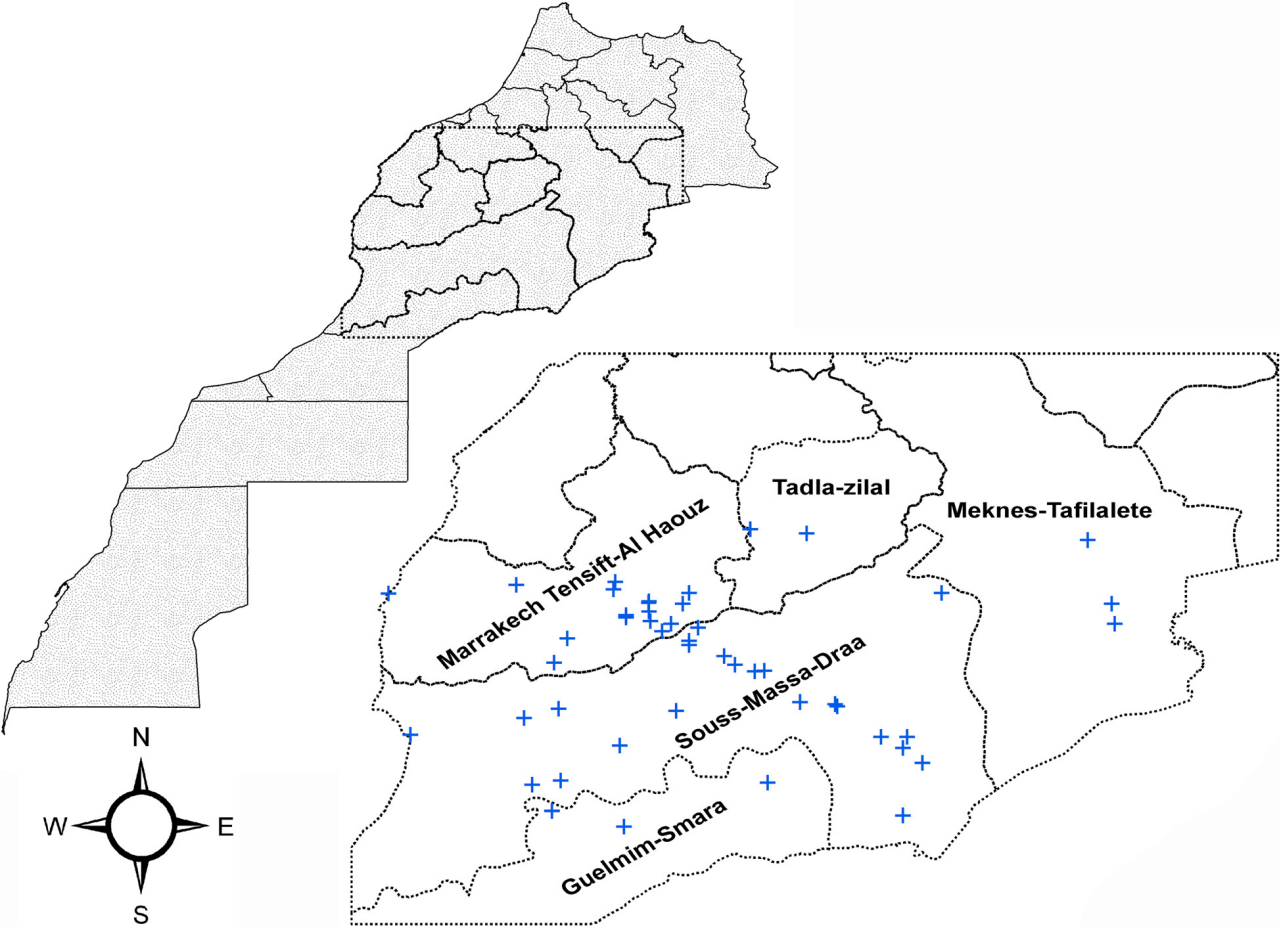
Regional and local map of the study sites. Dashed rectangle indicates the region sampled across Morocco and a blue cross reveals the sand fly sampling sites

Morocco is a northern African country, bordering the North Atlantic Ocean and the Mediterranean Sea. The current study covered sand fly sampling in 48 districts across central and southern Morocco (Fig. 1) with altitude ranges between sea level and up to 2400 m above sea. These districts represented five administrative regions: Guelmim-Es Smara, Marrakech-Tensift-Al Haouz, Meknes-Tafilalete, Souss-Massa-Draa and Tadla-Azilal. The climate in Morocco is mostly Mediterranean; however, seven bioclimatic regimes occur due to topographic differences across the country (e.g., high mountains, sub-humid, humid, semi-arid with cold winter, semi-arid with warm and temperate winter, arid and Saharan climate). The total population in Morocco is about 33,848,242 with an urbanization rate of 60.3 % [19].

### Sand fly collection and processing

Sand flies were collected from 48 districts across Morocco to include most foci identified as endemic in regard to CL transmission. Sand flies were collected by the sticky paper traps during three successive months of 2013 (i.e. April–June). The selection of this sampling period was in light of the previous observations reported from Morocco; (1) April-June corresponds to the highest peak of the sand fly abundance in Morocco [20, 21], and (2) high incidence of CL cases in this time interval annually [16, 22]. Sampling was carried out in rural areas around the human population (e.g., intradomestic, peridomestic, stables, barbacanes, old walls and rock crevices sites). The sticky traps were distributed so that each site had 30 traps/night (3 nights/site), with a total number of 4320 traps in 48 sites. Sticky traps were set at 6 pm and collected around 6 am of the next day. The trapped sand flies were collected in plastic bags coded with location, GIS coordinates, and date and transferred to the Laboratory of Ecology and Environment in Marrakesh, Morocco for further processing and identification.

In the laboratory, specimens were placed in 70 % ethanol until mounting. These specimens were then cleared in Mark André medium [23] and mounted on glass slides in Hoyer medium [24] prior to identification using the local morphological keys [25, 26]. The identification was based on the morphology of diagnostic characters including pharyngeal armature, female spermathecae and male genitalia. *P. sergenti* species identity was confirmed based on the morphological characters described in a previous study [27]. Species and forms of *Phlebotomus perniciosus* complex [28] were identified via diagnostic morphological characters; dilatation of the distal parts of the spermathecal ducts in females [29] and morphology of the copulatory valves and number of coxite hairs in males [30–32]. Sand fly species evenness and Bray-Curtis similarity between all sites were calculated using BioDiversity Professional statistics analysis software [33]. Finally, sand fly density was calculated based on the number of sand flies/m^2^ of sticky traps/night [20] to examine its relationship to the environmental changes across sampled sites.

### Environmental data

The study used several climatic, greenness, topographic and soil variables to examine the relationship between these environmental factors and the abundance of two potential vectors associated with circulation of CL across the country. These variables were obtained from different sources; (1) Land Surface Temperature (LST) and Normalized Difference Vegetation Index (NDVI) were downloaded from the Moderate Resolution Imaging Spectroradiometer (MODIS) satellite imagery for April to June 2013 corresponding to the same time of our collection. These data are available from Land Processes Distributed Active Archive Center obtained through the NASA Reverb Echo data portal (http://reverb.echo.nasa.gov/reverb). Maximum, minimum and mean of day-time temperature, night-time temperature and NDVI were calculated as described elsewhere [17, 34] for each grid cell across the entire study period so that a final set of 9 variables were used. (2) Precipitation data were obtained from the National Oceanic and Atmospheric Administration (NOAA) National Weather Center (http://www.cpc.noaa.gov). The precipitation data is based on daily estimates across the study area. We calculated the maximum and mean estimates of precipitation for April-June across each sampling site. (3) Aridity index, and soil water stress were downloaded from Consortium for Spatial Information (CGIAR*-*CSI; http://www.cgiar-csi.org/). (4) Elevation data were obtained from the Shuttle Radar Topography Mission (SRTM; http://srtm.usgs.gov/). (5) Soil data were obtained from the International Soil Reference and Information Centre (ISRIC) via the link (http://www.isric.org) to summarize chemical and physical soil characteristics across Morocco. These data represent the soil values in diverse depths across points sampled for soil properties, so, we calculated the mean of depths for the same variable. We used 5 variables reflecting the PH, bulk density, silt, clay and sand contents.

In sum, we have a set of 19 variables representing different climatic, topographic, greenness and soil variables. The values of each variable were extracted for each of 48 sampling sites and based on their digital coordinates using ArcGIS 10.3 (Environmental Systems Resource Institute, Redlands, California). The selection of these environmental variables was based on their direct and indirect influences on ecology and distribution of the sand fly populations [15, 35, 36].

### Data analysis

Data were analyzed using Minitab Version 17.1.0 [37]. An ANOVA test was used to compare the collections in different sites across the study area. Chi-squared test was used to test the deviation from the expected sex ratio equilibrium (1: 1). The correlations between sand fly densities and each of climate, elevation, greenness and soil variables were assessed using Pearson’s (*r*) correlation coefficient. Differences were considered significant only if *P* < 0.05.

## Results

### Sand fly species composition and sex ratios

A total of 11,717 sand flies were collected from 48 districts representing five governorates across Morocco, during April-June 2013 (Additional file 1; Table 1). The collected flies represented 11 species belonging to two genera (*Phlebotomus* and *Sergentomyia*); *Sergentomyia minuta* (*N* = 2653), *S. fallax* (*N* = 2053)*, P. perniciosus* (*N* = 1688), *P. papatasi* (*N* = 1631), *P. sergenti* (*N* = 1541), *P. longicuspis* (*N* = 1390), *P. ariasi* (*N* = 334), *S. dreyfussi* (*N* = 189), *P. alexandri* (*N* = 177), *S. africana* (*N* = 39), *P. chabaudi* (*N* = 22). *S. minuta* was the most predominant of the all collected flies; however, *P. perniciosus* was the dominant species of the genus *Phlebotomus*. There was a significant difference in sand fly collections across sampled sites (*P* = 0.00). No morphological anomalies were observed in the specimens of all collected species, except that *P. perniciosus* was collected only as atypical form (PNA).

**Table 1:** Phlebotomine Sand flies collected by sticky paper traps in several districts across Morocco from April to June 2013. These districts represent the cutaneous leishmaniasis endemic areas in Morocco

Species	Number of collected flies (F : M)						
	Guelmim-Smara	Marrakech-Tensift-Al Haouz	Meknes-Tafilalete	Souss-Massa-Draa	Tadla-zilal	Total	Ratio (F : M)
*Phlebotomus sergenti*	18/103	167/557	4/17	90/523	17/45	296/1245	1:4.2
*Phlebotomus papatasi*	57/174	98/425	19/106	166/572	3/11	343/1288	1:3.75
*Phlebotomus perniciosus*	2/15	372/1141	0/0	15/67	29/47	418/1270	1:3.03
*Phlebotomus longicuspis*	8/22	158/509	0/5	182/473	11/22	359/1031	1:2.87
*Phlebotomus ariasi*	0/4	103/193	0/0	5/29	0/0	108/226	1:2.09
*Phlebotomus alexandri*	1/22	4/31	6/56	13/44	0/0	24/153	1:6.37
*Phlebotomus chabaudi*	0/16	0/0	0/3	0/3	0/0	0/22	0
*Sergentomyia minuta*	16/102	477/1098	2/5	97/277	223/356	815/1838	1:2.25
*Sergentomyia fallax*	3/22	359/918	9/34	162/409	31/106	564/1489	1:2.64
*Sergentomyia* *dreyfussi*	2/16	28/85	2/4	19/33	0/0	51/138	1:2.70
*Sergentomyia* *africana*	7/14	0/0	1/11	1/5	0/0	9/30	1:3.33
Total	114/510	1766/4957	43/241	750/2435	314/587	2987/8730	1:2.92

The sex ratios (Females: Males) showed that overall collected males were approximately three times more than females (F/M = 1: 2.92; Table 1). Chi-squared test revealed a significant difference between the overall sex ratio and the expected sex ratio equilibrium (1: 1) for all species (*P* = 0.00).

### Sand fly diversity across sampling sites

Species evenness revealed differences in sand fly diversity among the different sampling sites with the highest overall diversity in Tagounite (J’ = 0.986) and the least diversity in Abadou where six species only occurred (J’ = 0.328). The species evenness and dendrogram of Bray-Curtis similarity are shown for comparison between sites in the supplementary materials (Additional file 2 and Additional file 3).

### Effect of environment on the distribution of CL vectors

**Fig. 2:**
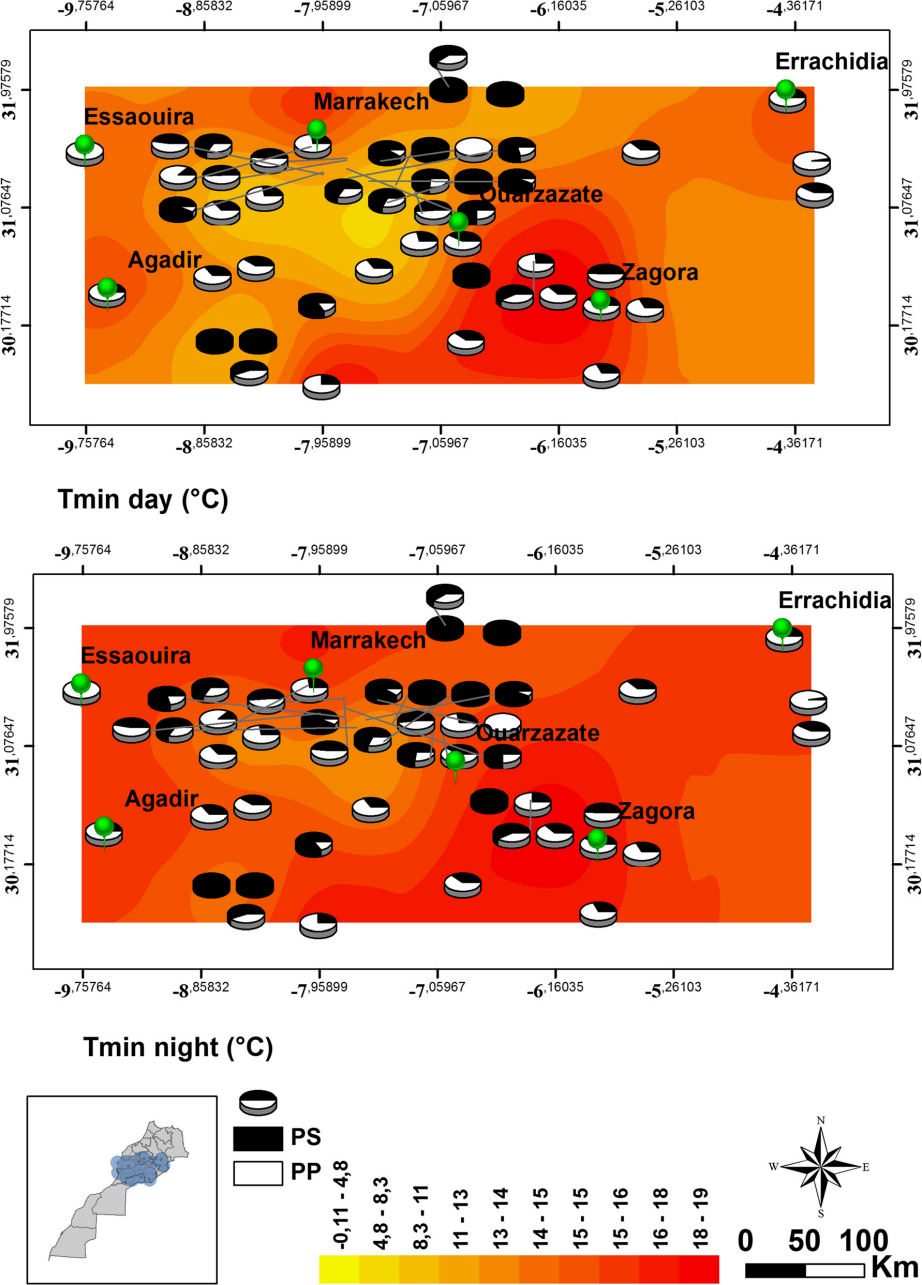
Densities of *Phlebotomus papatasi* (PP) and *P. sergenti* (PS) in 48 sites sampled across Morocco in relation to the changes in day-time and night-time minimum temperature

**Fig. 3:**
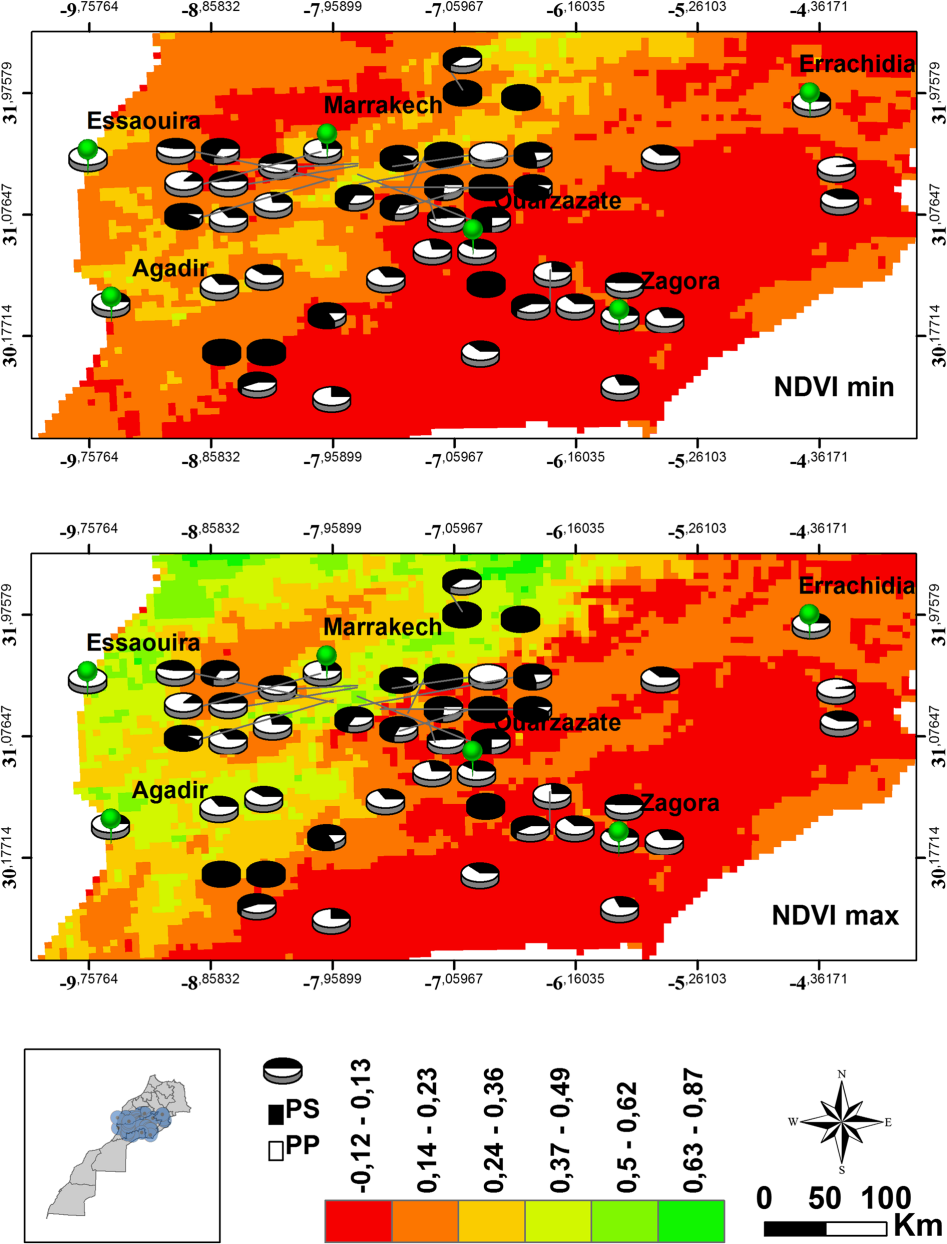
Densities of *Phlebotomus papatasi* (PP) and *P. sergenti* (PS) in 48 sites sampled across Morocco in relation to the changes in minimum and maximum normalized difference vegetation index

*P. papatasi* and *P. sergenti* co-occurred in 39 out of 48 sites sampled across Morocco. There was a difference between the densities of the *P. papatasi* and *P. sergenti* among the different sampling sites in regard to temperature and NDVI (Figs. 2 and 3). The correlations between abundance of *P. papatasi* and *P. sergenti* with several environmental factors were examined to understand the possible effect of these variables on the dynamics of these two vectors across the country (Table 2). The correlation of males and females differed in regard to each environmental condition. The densities of males and females *P. papatasi* were the most affected by temperature changes across all sampling sites. The study showed a significant positive correlation between the densities of both sexes of *P. papatasi* and night-time temperatures; however, no significant correlations were found with the day-time temperatures (Table 2). On the other hand, both sexes of *P. sergenti* showed no significant correlation with both day-time and night-time temperatures.

**Table 2:** Correlations of climatic, elevation, soil and vegetation index with the sand fly vector densities in 48 stations across Morocco. The correlations examined the relationship between male and female *Phlebotomus papatasi* and *Phlebotomus sergenti* densities to these environmental factors. Pearson correlation coefficient is presented in the table with *P* values between brackets

Variable	*P. papatasi*		*P. sergenti*	
	Male	Female	Male	Female
Climate				
Minimum temperature (night-time)	0.289 (0.047)^a^	0.315 (0.029)^a^	−0.005 (0.97)	0.001 (0.994)
Maximum temperature (night-time)	0.387 (0.007)^a^	0.423 (0.003)^a^	0.133 (0.368)	0.017 (0.909)
Mean temperature (night-time)	0.385 (0.007)^a^	0.406 (0.004)^a^	0.082 (0.58)	0.011 (0.942)
Minimum temperature (day-time)	0.304 (0.035)^a^	0.425 (0.003)^a^	0.008 (0.958)	−0.164 (0.267)
Maximum temperature (day-time)	0.127 (0.391)	0.257 (0.077)	−0.006 (0.967)	−0.139 (0.346)
Mean temperature (day-time)	0.204 (0.165)	0.338 (0.019)^a^	0.001 (0.996)	−0.162 (0.272)
Maximum precipitation	−0.224 (0.126)	−0.181 (0.219)	−0.075 (0.610)	−0.082 (0.580)
Mean precipitation	−0.134 (0.364)	−0.115 (0.436)	−0.005 (0.974)	−0.110 (0.458)
Mean aridity	−0.402 (0.005)^a^	−0.369 (0.010)^a^	−0.106 (0.473)	−0.018 (0.903)
Greenness				
Minimum NDVI	−0.064 (0.663)	−0.164 (0.264)	0.167 (0.258)	0.365 (0.011)^a^
Maximum NDVI	−0.137 (0.352)	−0.226 (0.123)	0.095 (0.521)	0.296 (0.04)^a^
Mean NDVI	−0.096 (0.515)	−0.189 (0.199)	0.130 (0.379)	0.335 (0.020)^a^
Altitude				
Altitude	−0.192 (0.190)	−0.171 (0.244)	0.059 (0.693)	0.009 (0.954)
Soil				
PH	−0.172 (0.241)	−0.412 (0.004)^a^	−0.193 (0.189)	0.022 (0.882)
Silt content	0.002 (0.989)	−0.121 (0.414)	0.049 (0.741)	−0.003 (0.985)
Clay content	0.007 (0.964)	−0.159 (0.279)	0.074 (0.619)	0.019 (0.895)
Sand content	−0.018 (0.905)	−0.168 (0.255)	0.021 (0.887)	−0.005 (0.975)
Bulk density	0.022 (0.880)	−0.134 (0.365)	0.062 (0.673)	0.021 (0.886)
Soil water stress	−0.399 (0.005)^a^	−0.372 (0.009)^a^	−0.103 (0.485)	−0.019 (0.900)

^a^ *significance level* (α) = 0.05

**Fig. 4:**
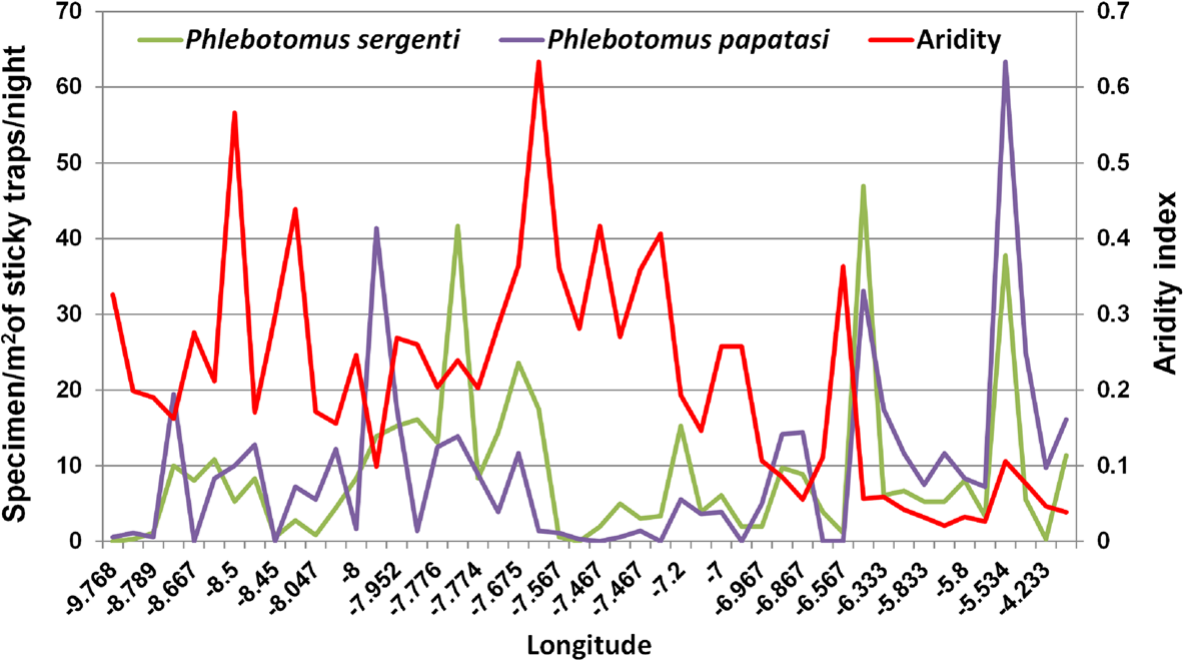
Fluctuation of sand fly densities in response to changes in aridity index across Morocco. The longitude of the sampling sites is presented on the horizontal axis

Male and female densities of both *P. papatasi* and *P. sergenti* showed negative correlation with the increase in precipitation; however, this correlation was not significant for both species. The densities of both species sexes showed negative correlation with aridity, but, this negative correlation was only significant in case of *P. papatasi* (Fig. 4; Table 2).

NDVI showed positive correlation with densities of *P. sergenti* males and females (Table 2), but a decrease in density of both sexes of *P. papatasi* was observed with increase of NDVI values. The correlation of NDVI and sand fly density was only significant in case of *P. sergenti* females.

*P. papatasi* was collected between 14 m and 1800 m altitude, while, *P. sergenti* was collected between 28 m and 1300 m altitude. The higher *P. papatasi* density was 63.33 specimens/m^2^/night in 1340 m altitude, whereas, *P. sergenti* was peaked with a density of 46.94 specimens/m^2^/night in 920 m altitude. The densities of males and females *P. papatasi* showed a negative correlation (*r* = −0.192, and −0.171, respectively) with altitude. However; *P. sergenti* was positively correlated with the increase in altitude but none of the correlations with altitude were significant for both species (*P* > 0.05; Table 2).

Finally, the correlation between densities of *P. papatasi* and *P. sergenti* and soil variables were also examined. Soil PH was negatively correlated with the density of female *P. papatasi* (*r* = −0.412, *P* = 0.004), while, soil water stress was negatively correlated with the male (*r* = −0.399, *P* = 0.005) and female (*r* = −0.372, *P* = 0.009) densities of *P. papatasi* (Table 2).

## Discussion

The main objective of this study was raised in response to our previous observations for site specific differences in sand fly collections. These observations led to a conclusion that environmental conditions available for each species could be different across Morocco which is exposed to continuous climate changes because of its unique location [15, 20]. We developed our current study to assess the relationship between the environmental conditions and the distribution of sand flies across endemic areas of CL. We also tried to examine the similarity between habitats in regard to the sand fly abundance. These results should be of importance to understand the environmental risk factors associated with high prevalence and establishment of vector populations for better understanding and guiding the target control programs in the country.

In this study, we included most CL endemic sites across the country to update sand fly fauna in all endemic sites and build a more comprehensive database for the abundance of two major vector species; *P. sergenti* responsible for transmission of ACL and *P. papatasi* responsible for transmission of ZCL among hosts. ACL is widespread in Guelmim-Es Smara, Marrakech-Tensift-Al Haouz, Souss-Massa-Draa and Tadla-Azilal regions such as Azilal, and Al Haouz and Chichaoua [22, 38, 39]. ZCL extended to the Saharan and pre-Saharan area of Guelmim-Es Smara, Meknes-Tafilalete and Souss-Massa-Draa regions such as Tata, Ouarzazate and Errachidia [4, 10]. The current study identified 11 species of sand flies in all habitats sampled; all of these species were previously identified in several other studies in Morocco [20–22, 31]. Sex ratios revealed more males in our collections; this result was not surprising given that the collection used sticky traps and occurred near the breeding sites of the sand flies. Sex ratios were also identified in favor of males in previous study in the region [40]. In the study area, *P. sergenti* and *P. papatasi* co-existed and shared equally the territory (51.41 and 48.58 % for *P. papatasi* and *P. sergenti*, respectively). In spite of this co-occurrence, their abundance and densities varied according to several ecological factors.

Previous studies revealed that adult sand flies could not survive outside the temperature range of 10–40 °C [41] while reproduction is not possible below 15 °C [42]. At around 30–32 °C, a significant increase in the vectorial capacity of sand flies was observed owing to shortening of the incubation period, despite a decrease in the vector’s survival [43]. In arid areas of Morocco, *P. papatasi* is most active in the hot, dry season and it was the most abundant species when ambient temperature is in the 32–36 °C range [20]. In southwest Asia, *P. papatasi* is most abundant in areas with a mean minimum temperature of 16 °C and mean maximum temperature of 44 °C from May to October [41].

In the present study, *P. papatasi* abundance seemed to be controlled mainly by the minimum temperature. Previous studies showed that ZCL (transmitted by *P. papatasi*) is prevalent in the pre-Saharan zones of North Africa where incidence and seasonal dynamic appeared to be controlled by minimum temperatures [16]. These similarities revealed the importance of minimum temperature to limit the distribution of both vector and disease spread in Morocco. This study noted also the significance of only night-time temperatures with *P. papatasi* abundance which was not surprising considering that sand flies have nocturnal habit and are found active especially during night.

One of the limiting factors for the distribution of disease vectors is vegetation. For example, sand fly males are attracted to plants as a source of sugar meals. Plants also provide suitable resting, breeding and mating sites [44]. Greenness, expressed in Normalized Vegetation index (NDVI), influences sand fly spatial repartition [16]. Examining sand fly density according to NDVI index may help with understanding the relationships between these species and vegetation. Here, NDVI index seemed to be a key determinant for *P. sergenti* distribution and abundance, especially females which were positively sensitive to NDVI variations.

In our study area, precipitation was not a determining factor for presence and abundance of these two potential CL vectors; the study was conducted in dry season and few changes in precipitation were reported. A negative correlation was reported in the study area between precipitation and density of both *P. papatasi* and *P. sergenti*; this observation reflected the high abundance of the same species in low precipitation reported early by another report in Morocco [20]. In contrast, aridity index, expressed as a function of rainfall and temperature, is one of the key factors negatively influencing the species density especially in the case of *P. papatasi* density. In Morocco, *P. papatasi* was found well adapted to arid climate conditions [14, 45], whereas, *P. sergenti* density was increased significantly in the arid and Saharan zones [14, 46]. Our results can also explain the absence of CL in the extreme southern Morocco which is an area characterized by higher aridity.

Many authors highlighted the role of soil conditions in spatial repartition of sand flies and also for characterization of sand fly breeding sites [47, 48]. Previous studies revealed that a combination of soil conditions, vegetation and topography create microclimates and ecological niches for *P. papatasi* [44]. Soil moisture was identified as an important limiting factor for the distribution of phlebotomine sand flies including *P. papatasi* [49, 50]. In the present study, soil water stress influences negatively both male and female *P. papatasi* densities. Soil water stress is the monthly fraction of soil water content available for evapotranspiration and is inversely proportional to maximum soil moisture content, so a decrease in the soil water stress is caused by high soil moisture which is favorable conditions for an increase in the sand fly abundance. Similar observations for this increase in the sand fly densities were observed in other countries [51]. This latter study identified a positive correlation between soil moisture and *P. papatasi* density. Schlein et al. [49] revealed that scarcity of water was the key-factor that limit the sand fly abundance and spread of leishmaniasis across Israel. However, the available sources of water moisture may be different between endemic sites across the Middle East including Morocco but soil moisture remain important for maintaining *P. papatasi* populations in all habitats including arid desert areas where irrigation activities occur.

The combination of the effect of several environmental factors determines the vector distribution. Our results revealed some of these variables and support the previous studies regarding the importance of soil moisture as one of the key factors that limit the distribution of the sand flies across the region. So, any ecological studies that focus on mapping vector species should consider these variables as important parameters in their studies. This study reflects a huge sampling across Morocco but we plan to further extend our current studies to include two more dimensions as our first priorities; (1) to include several other species associated with other forms of leishmaniasis, (2) to include long-term surveillance which examines the contribution of these variables to the vector species and disease dynamics for detailed insights on the long-term maintenance of disease hotspots across Morocco. Finally, one of the most important further objectives of this study is to explore mapping exercises and habitats suitability of CL vectors across the country to guide our surveillance programs.

## Conclusion

The results of the present study suggest preliminary landmarks of vector distribution and CL risk in Morocco. The climatic factors (night-time temperature and aridity) with soil conditions and NDVI index are important factors to anticipate areas of disease risk and characterize areas of surveillance priorities for ZCL and ACL to guide the efforts for successful vector surveillance and control programs.

## Additional files

Additional file 1:
**Detailed data set for sand fly species sampled in 48 sites across Morocco from April to June 2013.** The specimens were sorted as male or female for each species. The coordinates (longitude and latitude) for each site is also presented in this data set. (XLSX 23 kb) Additional file 2:
**Species evenness across 48 sampling sites in Morocco.** The Species evenness is calculated using the BioDiversity Professional statistics analysis software [33]. (DOCX 14 kb) Additional file 3:
**Bray-Curtis similarity based on the sand fly abundance across all sampling sites in Morocco.** The scale represents the similarity between the sampling sites regarding the sand fly abundance. (DOC 115 kb)
