# Supplementary material for: Species composition of sand flies and bionomics of Phlebotomus papatasi and P. sergenti (Diptera: Psychodidae) in cutaneous leishmaniasis endemic foci, Morocco
Source: Parasit Vectors. 2016 Feb 2;9:60. doi: 10.1186/s13071-016-1343-6 (PMC4736259; doi:10.1186/s13071-016-1343-6)
Supplement: Additional file 2: — Species evenness across 48 sampling sites in Morocco. The Species evenness is calculated using the BioDiversity Professional statistics analysis software [33]. (DOCX 14 kb) [file 13071_2016_1343_MOESM2_ESM.docx]

**Additional file 2: S**pecies evenness across 48 sampling sites in Morocco. The Species evenness is calculated using the BioDiversity Professional statistics analysis software [[33](#_ENREF_31)].

| Locality | Evenness |
| --- | --- |
| Zaouit Bouhouta | 0.839 |
| Tahnaoute | 0.734 |
| Yabora | 0.729 |
| Lkhmis | 0.747 |
| Setti Fadma | 0.794 |
| Aagreb | 0.633 |
| Oulad Yahia | 0.828 |
| Abadou | 0.328 |
| Lhronna | 0.664 |
| Imi n’Ifri | 0.57 |
| Damnat | 0.65 |
| Tanant | 0.691 |
| Azilal | 0.64 |
| Sidi Baghdad | 0.979 |
| Ait ouaarab | 0.836 |
| Ait belabass | 0.88 |
| El Hajeb | 0.972 |
| Marrakech | 0.941 |
| Touama | 0.777 |
| Douar Igmir | 0.568 |
| Arghal | 0.653 |
| Aguelmous | 0.844 |
| Agouim | 0.801 |
| Tagouimat | 0.704 |
| Amezgan | 0.674 |
| Tabourihit | 0.617 |
| Fedragon | 0.788 |
| Ouarzazate | 0.808 |
| Chichaoua | 0.883 |
| Agadir | 0.869 |
| Essaouia | 0.865 |
| Tinghir | 0.645 |
| Tisserghate | 0.723 |
| Tinzouline | 0.765 |
| Beni Zoli | 0.954 |
| Zagora | 0.834 |
| Anagam | 0.849 |
| Tagounite | 0.986 |
| M'hamid | 0.846 |
| Foum Zguid | 0.812 |
| Tata | 0.696 |
| Issafn | 0.922 |
| Igherm | 0.624 |
| Ouled Berhil | 0.82 |
| Tinmel | 0.814 |
| Ouirgane | 0.868 |
| Erfoud | 0.744 |
| Errachidia | 0.697 |
