# Supplementary material for: Species composition of sand flies and bionomics of Phlebotomus papatasi and P. sergenti (Diptera: Psychodidae) in cutaneous leishmaniasis endemic foci, Morocco
Source: Parasit Vectors. 2016 Feb 2;9:60. doi: 10.1186/s13071-016-1343-6 (PMC4736259; doi:10.1186/s13071-016-1343-6)

**Additional file 3:** Bray-Curtis similarity based on the sand fly abundance across all sampling sites in Morocco. The scale represents the similarity between the sampling sites regarding the sand fly abundance.


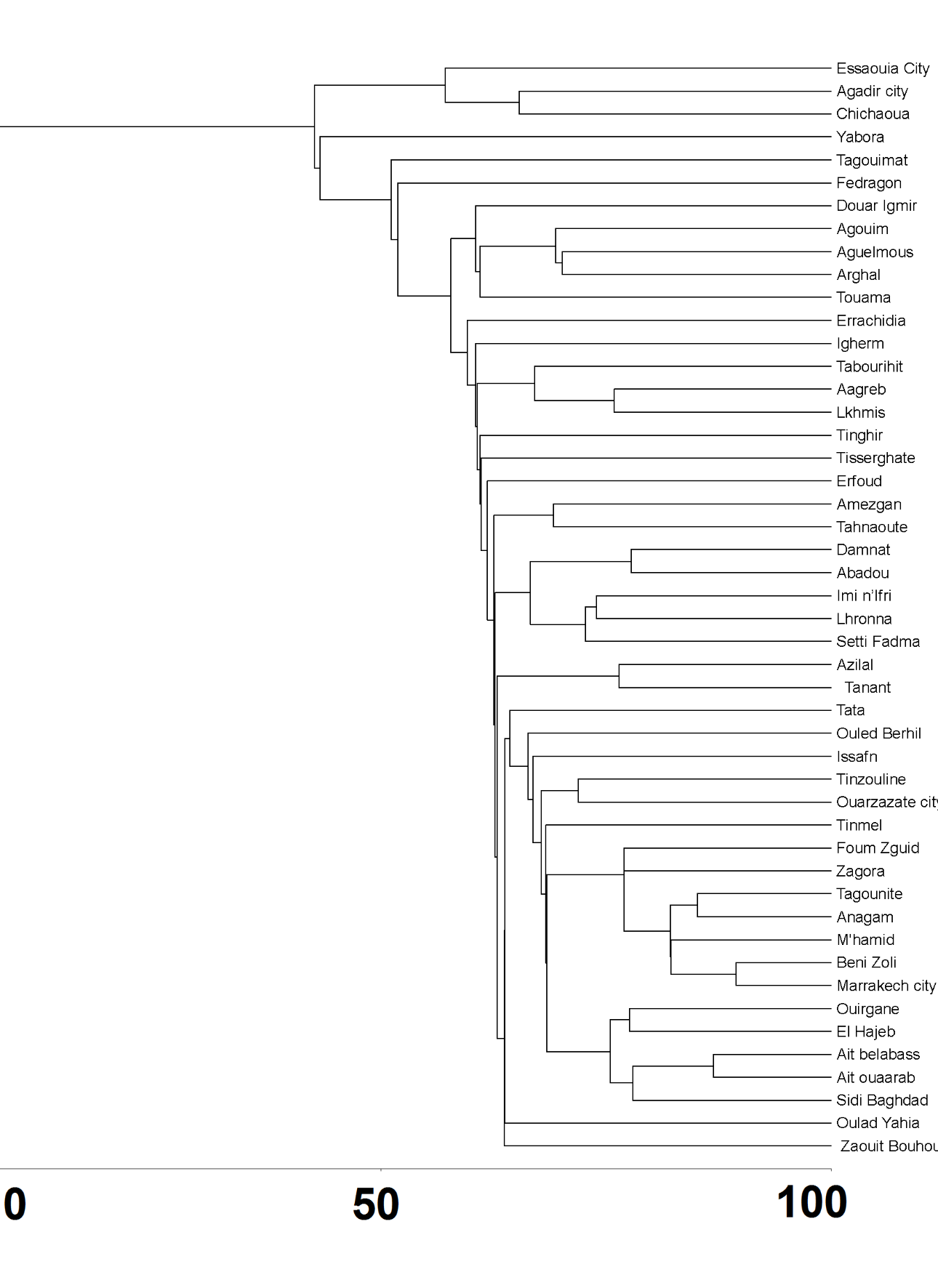

Supplement: Additional file 3: — Bray-Curtis similarity based on the sand fly abundance across all sampling sites in Morocco. The scale represents the similarity between the sampling sites regarding the sand fly abundance. (DOC 115 kb) [file 13071_2016_1343_MOESM3_ESM.doc]
